# A Cross-Sectional Study of Ageing and Cardiovascular Function over the Baboon Lifespan

**DOI:** 10.1371/journal.pone.0159576

**Published:** 2016-07-18

**Authors:** Kristen R. Yeung, Christine L. Chiu, Suzanne Pears, Scott J. Heffernan, Angela Makris, Annemarie Hennessy, Joanne M. Lind

**Affiliations:** 1 Western Sydney University, School of Medicine, Sydney, Australia; 2 Department of Renal Medicine, Royal Prince Alfred Hospital, Sydney, Australia; 3 The Heart Research Institute, Sydney, Australia; 4 Nephrology Department, Liverpool Hospital, Sydney, Australia; The University of Manchester, UNITED KINGDOM

## Abstract

**Background:**

Ageing is associated with changes at the molecular and cellular level that can alter cardiovascular function and ultimately lead to disease. The baboon is an ideal model for studying ageing due to the similarities in genetic, anatomical, physiological and biochemical characteristics with humans. The aim of this cross-sectional study was to investigate the changes in cardiovascular profile of baboons over the course of their lifespan.

**Methods:**

Data were collected from 109 healthy baboons (*Papio hamadryas*) at the Australian National Baboon Colony. A linear regression model, adjusting for sex, was used to analyse the association between age and markers of ageing with P < 0.01 considered significant.

**Results:**

Male (n = 49, 1.5–28.5 years) and female (n = 60, 1.8–24.6 years) baboons were included in the study. Age was significantly correlated with systolic (R^2^ = 0.23, P < 0.001) and diastolic blood pressure (R^2^ = 0.44, P < 0.001), with blood pressure increasing with age. Age was also highly correlated with core augmentation index (R^2^ = 0.17, P < 0.001) and core pulse pressure (R^2^ = 0.30, P < 0.001). Creatinine and urea were significantly higher in older animals compared to young animals (P < 0.001 for both). Older animals (>12 years) had significantly shorter telomeres when compared to younger (<3 years) baboons (P = 0.001).

**Conclusion:**

This study is the first to demonstrate that cardiovascular function alters with age in the baboon. This research identifies similarities within cardiovascular parameters between humans and baboon even though the length of life differs between the two species.

## Introduction

Ageing is a complex biological process in humans that leads to well-defined phenotypic changes in physiological and metabolic functioning [1], that render the cardiovascular system prone to disease. The prevalence of cardiovascular diseases, such as hypertension and heart disease, increase as individuals age [2–4]. However, it is difficult to determine what contribution the ageing process has to the development of cardiovascular disease in the elderly, and to what extent a poor lifestyle accounts for these conditions [5–11].

Baboons are an ideal translational model of human ageing. Unlike rodent animal models, the baboon exhibits a greater degree of similarity to humans in regards to genetic, anatomical, physiological and biochemical characteristics [12–14]. The baboon has been used extensively as an animal model of human disease [14], including studies on markers of physical capability [15].

Markers of cardiovascular function can be used to assess the extent of biological ageing in an individual. This includes the measurement of oxidative stress via the production of reactive oxygen species, inflammation, blood pressure and arterial stiffness as indicated by pulse wave dynamics and increases in pulse pressure [16]. Telomere length has also been widely considered as a marker for biological ageing [17]. However, it has not been established whether the dynamics of telomere attrition *in vivo* has a major role in the biology of human ageing or is purely a consequence of the ageing process [18]. [12]Telomere shortening and cellular ageing appear to be highly conserved among primates [19]. Thus, the baboon is a suitable model for studying the cardiovascular profile and effects of ageing, as there is increased relevance when translating findings to humans. Baboons are known to develop atherosclerotic lesions with models of experimental atherosclerosis, in the presence of moderate hyperlipidemia, having clinical characteristics similar to that seen in the early stages of human disease [20]. Captive baboons are also valuable for use in cardiovascular and ageing research as environmental variation (including diet) can be controlled to some extent.

Little is known about whether non-human primates have a similar pattern of ageing across their lifespan. No studies that have comprehensively investigated traditional markers of ageing in the baboon including serum biochemistry, pulse wave dynamics and telomere length. Understanding how these markers vary at different stages of baboon development will help to determine which markers occur as a result of the natural ageing process even within a shorten time-span, and minimising the environmental lifestyle factors that confound ageing research in humans. [21]

The aim of this cross-sectional study was to investigate the changes in cardiovascular profile and telomere length in a captive colony of baboons where diet and environment are controlled over the course of their lifespan.

## Methods

Experiments were approved by the Sydney Local Health District Animal Welfare Committee (SSWAHS 2001/025) and ratified by the Western Sydney University Animal Ethics Committee (A9758). Care of the animals was conducted in accordance with the Australian National Health and Medical Council’s (NHMRC) Code of Practice for the Care and Use of Non-Human Primates for Scientific Purposes.

The animals used in this study were baboons (*Papio hamadryas*) from the Australian National NHMRC Baboon Colony (Sydney Australia), which was established in 1982. A closed breeding programme is in place, with no outside animals introduced to the colony. Baboons are housed in one male units (OMUs), with between four and seven females per male, reflecting the social organization unique among the baboon species to *Papio hamadryas*. The complex of outdoor enclosures maintains the troop structure of the colony by allowing visual and vocal contact. They are provided with visual and auditory barriers and shelter which they can freely access. They can also freely access an indoor night house which mimics a rock wall for sleeping.

The husbandry practices at the colony consist of daily cage cleaning, twice daily feeding and, annual health screening. The diet consists of fresh fruit and vegetables, bread, nuts, sunflower seeds, and commercial primate pellets. Fresh water is provided *ad libitum*. Various forms of enrichment are provided to the animals including logs, tree branches, swings, water features, and mirrors all of which are permanent features within or on the outside of the cages. Baboons are also strongly motivated by food which makes up a large part of additional enrichment given throughout the week. This includes seed and nut tubes, fruit and vegetable iceblocks, and food puzzles.

Health screening includes physical examination, tuberculin testing, tetanus vaccination, intestinal parasite control, and sample collection for routine biochemical, haematological, and microbiological studies

Data were collected from 109 baboons during the colony annual health check. Animals included in this study were healthy male and non-pregnant female baboons. Animals were grouped based on physiological changes that occur with age; juveniles (<3 years), adolescents (3–7 years), young adults (7–12 years), and older animals (>12 years). All data were collected from animals during their colony annual health check. Measurements were obtained from anaesthetised baboons as intensive behavioural training is required to undertake conscious blood pressure readings. Animals were anaesthetised using intramuscular ketamine (8mg/kg; Provet Laboratories Pty Ltd., Sydney, NSW, Australia) as previously described [22].

### Blood Pressure Measurement

Blood pressure measurements were obtained from anaesthetised baboons using an indirect cuff method, following the manufacturer’s instructions and recommended techniques [23]. After ensuring that a uniform and consistent depth of anaesthesia had been reached (no spontaneous movement of limbs, no vocalisation, no withdrawal response to finger pinch, and no eyelash reflex), the cuff of the automatic oscillometric device (Propaq LT monitor; Welch-Allyn, Beaverton, OR, USA) was placed on the right upper arm of the animal. Cuff width was approximately 40% of the limb circumference. Each animal had one blood pressure reading taken at two minute intervals, for a total of three measurements and the average recorded for systolic and diastolic blood pressure. Heart rate was measured using the automated oscillometric device.

### Pulse Wave Analysis

Assessment of arterial wave reflection characteristics was performed non-invasively using the SphygmoCor^®^ system (Atcor Medical, West Ryde, NSW, Australia). The radial artery pressure waveforms were recorded by applanation tonometry of the radial pulse in the right wrist using a micromanometer (Millar Instruments, Houston, TX, USA). The radial blood pressure and waveforms were calibrated from the systolic and diastolic brachial artery blood pressures. A generalised transfer function was applied to the radial artery waveform to derive the corresponding aortic pressure waveform. The human transform function was used as this is not available for primates. Aortic pressures, augmentation pressure, and augmentation index were calculated using the aortic pressure waveform. The augmentation pressure is defined as the height of the late systolic peak above the inflection point on the waveform and may be positive or negative depending on the relative heights of the two peaks. The augmentation index is defined as augmentation pressure expressed as a percentage of the aortic pulse pressure and will also be positive or negative depending on the augmentation pressure.

Each animal had three separate measurements collected at two minute intervals and the average recorded. All measurements were performed in the same room and collected by the same researcher. Only high quality recordings, defined as an operator index of > 80% were included in the analysis.

### Blood and Urine collection

Blood and urine samples were collected by a licensed veterinarian during the animal's routine annual health check. Blood was collected for biochemical analysis and genomic DNA extraction. Biochemical testing was performed at the Royal Prince Alfred Hospital specimen laboratory and included measurement of serum sodium (via the indirect ion selective electrodes method), creatinine (via the kinetic Jaffe reaction and measurement of alkaline picrate at 505 nm), albumin (measured via bromocresol green at 600 nm), total cholesterol (measured via the reaction between cholesterol esterase and cholesterol oxidase yielding 4-amino phenazone and phenol measured at 505 nm), triglycerides (measured via hydrolysis with lipase followed by enzymatic assays coupled with the Trinder reaction, measured at 505 nm) and urea (measured using the urease/GLDH rate reaction coupled to NADH, read at 340 nm). A spot urine sample was collected using a sterile catheter inserted into the urethra to measure urinary albumin (using Immulite XPi), urinary protein (using the automated turbidimetric method) and urinary creatinine concentrations (via the kinetic Jaffe reaction and measurement of alkaline picrate at 505 nm).

### Relative telomere length

Relative telomere length in leukocytes was measured using quantitative PCR as previously described [24]. A subset of animals was selected to establish a comparison between young and old (young 12 males and 12 females and old 8 females and 7 males). This method expresses telomere length as a ratio (T/S) of telomere copy number (T) to haemoglobin subunit beta (*HBB)* single copy gene (S) within each sample. Therefore, a higher T/S corresponds to longer telomeres. The PCR reactions were carried out under the following conditions 95°C– 10 min; 40 cycles (95°C– 30 sec; 60°C– 1 min); followed by a dissociation curve. All samples were run in triplicate. Cycle threshold (Ct) values for each sample were calculated using the MxPro QPCR software (Stratagene, Agilent Technologies, USA). Triplicate Ct values were averaged and the quantity of each sample was calculated using the delta-delta Ct method [25].

### Statistical analysis

Statistical analysis was performed with SPSS 22.0 (IBM, New York, NY, USA). A linear regression model was applied to the cardiovascular and biochemical parameters with age, adjusted for sex. A general linear model was used to compare T/S ratios between young and old animals. The significance level was set at P < 0.01 to account for multiple testing. Animals with missing data were excluded from the analysis of that variable.

## Results

This study measured markers of cardiovascular function in a captive colony of baboons (n = 109). Table 1 summarises descriptive characteristics of the cohort stratified by age and sex. A significant correlation between age and body weight was observed with increasing age associated with increasing weight (Table 2).

**Table 1:** Demographics of the cohort stratified by sex and age.

	n	Age (years)*	Weight (kg)*
**Males**			
Young (0 to < 3)	16	2.25 ± 0.52	6.41 ± 1.20
Adolescent (3 to < 7)	24	4.73 ± 1.02	16.25 ± 5.88
Young Adults (7.0 to <12)	2	8.63 ± 1.59	27.25 ± 1.06
Adults (> 12)	7	20.43 ± 3.94	22.27 ± 3.06
**Females**			
Young (0 to < 3)	17	2.39 ± 0.33	5.62 ± 1.00
Adolescent (3 to < 7)	29	4.48 ± 1.04	11.08 ± 2.15
Young Adults (7.0 to <12)	8	10.49 ± 0.83	13.79 ± 2.20
Adults (> 12)	6	21.71 ± 4.45	13.53 ± 1.69

*data shown as mean ± SD

**Table 2:** Correlation between cardiovascular and biochemical parameters with age (n = 109).

Variable	*Pearson correlation coefficient	Adj. R^2^	P value
Systolic blood pressure	0.49	0.23	<0.001^a^^b^
Diastolic blood pressure	0.66	0.44	<0.001^a^^b^
Heart rate	-0.16	0.12	0.05
Core augmented index	0.41	0.17	<0.001^a^^b^
Av. core pulse pressure	0.54	0.30	<0.001^a^^b^
Cholesterol	-0.29	0.07	0.002^a^
Creatinine	0.63	0.43	<0.001^a^^b^
Triglycerides	0.14	0.02	0.08
Urea	0.32	0.09	<0.001^b^
Serum sodium	-0.18	0.03	0.03^a^
Serum albumin	-0.10	-0.01	0.15
Urinary protein	0.16	0.02	0.05^b^
Urinary micro-albumin	0.13	<0.001	0.10

*regression analysis adjusted for sex;

^a^significant in males;

^b^significant in females

Linear regression was performed on a number of cardiovascular and biochemical parameters, with age (Table 2). Age was significantly correlated with systolic and diastolic blood pressure, with blood pressure increasing with age. Age was also significantly correlated with measures of endothelial function including core augmentation index and core pulse pressure. Older animals had reduced measures of endothelial function when compared with younger animals. There was no significant association between age and heart rate. Creatinine and urea increased with increasing age, while cholesterol levels decreased with increasing age. No associations between age and triglyceride, serum sodium, or albumin measures were observed. Similarly, no association between age and urinary protein or urinary microalbumin excretion were found (Table 2). For data stratified by sex see S1 Table.

**Fig 1:**
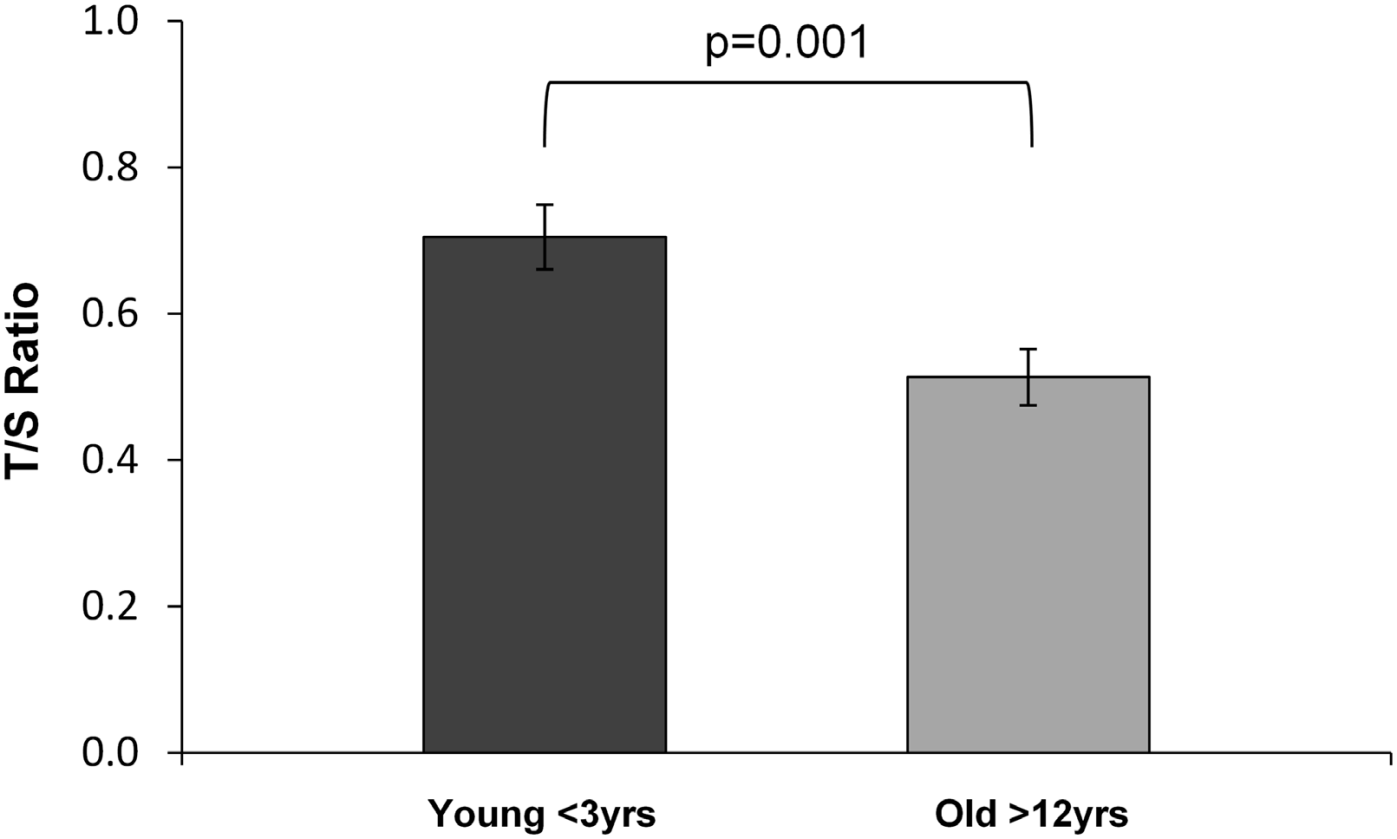
Relative telomere length in a subset of old (> 12 years) (n = 15) and young (< 3 years) (n = 24) animals. Telomere length is shown as a ratio (T/S) of telomere copy number (T) to the *HBB* single copy gene (S). Data are shown relative to the young animals, adjusted for sex, and expressed as mean ± SE.

Relative telomere length was measured in the oldest and youngest animals to establish a comparison between young and old. Age was inversely related to telomere length (Fig 1). Older animals had significantly shorter telomeres compared to young baboons, adjusting for sex (P = 0.001). Corresponding blood pressures were also significantly different between these two groups in this subset of animals. Older animals had significantly higher systolic blood pressure and diastolic blood pressure compared with young animals, adjusting for sex (P < 0.001).

## Discussion

This cross-sectional study measured the cardiovascular profile as a marker of biological ageing in a captive colony of baboons. We demonstrated declining cardiovascular function with age in healthy baboons living in a setting where the influences of the external environment and diet are relatively controlled. There was a significant increase in systolic and diastolic blood pressure with age along with an increased augmentation index and pulse pressures, indicative of cardiovascular function decline in older animals. Creatinine and urea measures were significantly increased in older animals and telomere length was significantly shorter in the older baboons. This study was carried out in healthy animals, demonstrating patterns associated with the ageing process.

A number of studies have used the non-human primate as a model to investigate age-related diseases such as diabetes and atherosclerosis, immune senescence, the visual system, and osteoarthritis [26–31]. To our knowledge, this is the first study to investigate the relationship between blood pressure, pulse wave dynamics, biochemical markers and age in the baboon.

In humans, studies indicate that there is an association between blood pressure and age in Western populations [2, 32, 33]. Conversely, studies that have investigated patterns of blood pressure in remote populations, report low average blood pressure and an absence or near absence of hypertension with age [5–9]. These populations are usually isolated and characterised by the absence of the stressors found in westernised cultures, higher levels of moderate to intense physical activity, and diets lower in salt [8]. The present study was conducted in a captive colony of baboons that live in a relatively stress-free environment, free from predators and with ample access to food and water. These animals consume a low fat, low salt diet similar to the studies conducted in remote human populations. Despite this we found that blood pressure increased with age in these healthy baboons.

This study also found a correlation between augmentation index and pulse pressure with age in the baboon, with older animals displaying higher values. Pulse wave analysis is a simple, non-invasive technique that provides data on the mechanical properties of the arterial tree including arterial stiffness and it can also be used to assess endothelial function [34, 35]. Augmentation index is a composite measure of aortic wave reflection and systemic arterial stiffness [36, 37]. The observed combination of increased pulse pressure and markers of arterial stiffening suggest a decline in cardiovascular function with age in the baboon.

A study investigating the relationship between age and blood chemistry and haematological variables in captive rhesus macaques reported differences in creatinine, albumin and calcium in ageing animals [38, 39]. In humans, lower levels of serum albumin have also been observed with aging, and are associated with loss of muscle mass [40, 41]. In the present study, no significant correlation between age and albumin levels was observed.

Serum creatinine levels were significantly increased with age in the present study, which is similar to results of human studies [42–45]. Increases in serum creatinine are known to indicate age-related changes in renal function including a reduced glomerular filtration rate and decreased renal blood flow [42, 46]. The results of the present study suggest that renal function declines with age in the baboon.

We found that cholesterol significantly decreased with age in the baboon. This is in contrast to previous work in humans, which has shown that elevated serum cholesterol and triglycerides are potential risk factors for cardiovascular diseases in the elderly [47, 48]. Further research is required to clarify why there were differences seen in the present study.

In addition to the physiological markers of ageing, this study demonstrated that leukocyte telomere length significantly decreases with age. Similar results have been reported in a study of 16 baboons aged 0.2 to 26.5 years, where telomere lengths in granulocytes and lymphocytes were significantly shorter in older animals [49]. The pattern of telomere length dynamics in leukocytes from baboons appears to be very similar to what has been previously described in humans [50]. Telomere shortening represents one molecular mechanism that occurs across a range of tissues during human ageing, and is considered a biomarker of physiological age, cumulative cell damage, and cardiovascular risk [51–54]. We have shown that cardiovascular decline occurs in ageing captive baboons and correlates to similar ageing events in humans.

There were some limitations to this study to be noted. All the baboons lived in essentially identical environments and were fed a controlled diet. However, the extent to which the environment could be controlled may have varied. The social construct of the *hamadryas* baboon involves a hierarchal structure and by nature some baboons may be situated in a comparatively higher stress environment depending on their rank in the social group. Younger animals are less likely to cope in these situations. In our study, younger animals demonstrated higher serum cholesterol levels compared to older animals, when the reverse has been demonstrated in humans, this may be one explanation for these observations. This study also had a higher proportion of younger animals, relative to older animals, due to the age range of the animals within the colony. The conclusions would have been strengthened with a larger proportion of older animals. It should also be noted that in this study animals were kept in family groups and fed together rather than given individual portions. Animals that were higher up in the social hierarchy would have had first access to food and thus there may have been some dietary differences between animals. Hierarchy analysis was not possible in the current study due to the constantly changing social hierarchy situations for these groups.

Despite these limitations, we have been able to complete a cross-sectional study to demonstrate age-related changes within a captive colony and thus the baboon has proven to be a valuable model for studying the cardiovascular profile across the lifespan. In this study, blood pressure increased with age and pulse wave characteristics were higher in older animals. Additionally, telomere length served as a potential indicator of biological ageing as older animals had significantly shorter telomeres compared to young animals. Given that the ageing process is multifactorial and highly variable, this study builds upon others that have proposed telomere length as a biomarker of ageing and this research also provides a new dimension to the study of cardiovascular function and decline. This research also demonstrates that these markers of ageing are evident within the aged baboon even though the baboon has a much shorter life-span than humans.

This research provides an enhanced understanding of the age-related physiological changes that occur in captive non-human primates, specifically the *Papio hamadryas* baboon. We have measured the cardiovascular profile in 109 baboons at different ages, and have shown that cardiovascular function alters with age in the baboon, with increases in blood pressure and arterial stiffening occurring. This serves as a baseline for the normal physiology that occurs with ageing. This research demonstrates that non-human primates may serve as models for the interplay between cardiovascular function and ageing in humans, further helping us to unravel the complex nature of the ageing process.

## Supporting Information

S1 Table Correlation between cardiovascular and biochemical parameters with age, stratified by sex. (DOCX)

## References

[1] Lara J, Godfrey A, Evans E, Heaven B, Brown LJ, Barron E, et al Towards measurement of the Healthy Ageing Phenotype in lifestyle-based intervention studies. *Maturitas*. 2013;76(2):189–99. 10.1016/j.maturitas.2013.07.007 23932426

[2] Ostchega Y, Dillon CF, Hughes JP, Carroll M, Yoon S. Trends in hypertension prevalence, awareness, treatment, and control in older U.S. adults: data from the National Health and Nutrition Examination Survey 1988 to 2004. *Journal of the American Geriatrics Society*. 2007;55(7):1056–65. . 17608879 10.1111/j.1532-5415.2007.01215.x

[3] Rosamond W, Flegal K, Furie K, Go A, Greenlund K, Haase N, et al Heart disease and stroke statistics—2008 update: a report from the American Heart Association Statistics Committee and Stroke Statistics Subcommittee. *Circulation*. 2008;117(4):e25–146. . 18086926 10.1161/CIRCULATIONAHA.107.187998

[4] Ungvari Z, Sonntag WE, Csiszar A. Mitochondria and aging in the vascular system. *Journal of molecular medicine*. 2010;88(10):1021–7. 10.1007/s00109-010-0667-5 20714704 PMC3045746

[5] Carvalho JJ, Baruzzi RG, Howard PF, Poulter N, Alpers MP, Franco LJ, et al Blood pressure in four remote populations in the INTERSALT Study. *Hypertension*. 1989;14(3):238–46. . 2767757 10.1161/01.hyp.14.3.238

[6] Dahl LK. Salt and hypertension. *The American journal of clinical nutrition*. 1972;25(2):231–44. . 5009786 10.1093/ajcn/25.2.231

[7] Lowenstein FW. Blood pressure in relation to age and sex in the tropics and subtropics. A review of the literature and an investigation in two tribes of Brazil indians. *The Lancet*. 1961;277(7173):389–92.

[8] Oliver WJ, Cohen EL, Neel JV. Blood pressure, sodium intake, and sodium related hormones in the Yanomamo Indians, a "no-salt" culture. *Circulation*. 1975;52(1):146–51. . 1132118 10.1161/01.cir.52.1.146

[9] Page LB, Damon A, Moellering RC Jr. Antecedents of cardiovascular disease in six Solomon Islands societies. *Circulation*. 1974;49(6):1132–46. . 4831656 10.1161/01.cir.49.6.1132

[10] Truswell AS, Kennelly BM, Hansen JD, Lee RB. Blood pressures of Kung bushmen in Northern Botswana. *American heart journal*. 1972;84(1):5–12. . 5080283 10.1016/0002-8703(72)90299-2

[11] Stamler J. The INTERSALT Study: background, methods, findings, and implications. *The American journal of clinical nutrition*. 1997;65(2 Suppl):626S–42S. . 9022559 10.1093/ajcn/65.2.626S

[12] Wang XL, Wang J, Shi Q, Carey KD, VandeBerg JL. Arterial wall-determined risk factors to vascular diseases: a nonhuman primate model. *Cell biochemistry and biophysics*. 2004;40(3):371–88. . 15211033 10.1385/CBB:40:3:371

[13] Willis EL, Wolf RF, White GL, McFarlane D. Age- and gender-associated changes in the concentrations of serum TGF-1beta, DHEA-S and IGF-1 in healthy captive baboons (Papio hamadryas anubis). *Gen Comp Endocrinol*. 2014;195:21–7. 10.1016/j.ygcen.2013.10.004 24161750 PMC3888644

[14] Jolly CJ. A proper study for mankind: Analogies from the Papionin monkeys and their implications for human evolution. *Am J Phys Anthropol*. 2001;Suppl 33:177–204. . 11786995 10.1002/ajpa.10021

[15] Huber HF, Gerow KG, Nathanielsz PW. Walking speed as an aging biomarker in baboons (Papio hamadryas). *J Med Primatol*. 2015;44(6):373–80. 10.1111/jmp.12199 26411922 PMC4802968

[16] Hubbard JM, Cohen HJ, Muss HB. Incorporating Biomarkers Into Cancer and Aging Research. *Journal of clinical oncology*: *official journal of the American Society of Clinical Oncology*. 2014;32(24):2611–6. . 25071114 10.1200/JCO.2014.55.4261 PMC4876339

[17] Blackburn EH. Switching and signaling at the telomere. *Cell*. 2001;106(6):661–73. . 11572773 10.1016/s0092-8674(01)00492-5

[18] Benetos A, Okuda K, Lajemi M, Kimura M, Thomas F, Skurnick J, et al Telomere length as an indicator of biological aging: the gender effect and relation with pulse pressure and pulse wave velocity. *Hypertension*. 2001;37(2 Pt 2):381–5. . 11230304 10.1161/01.hyp.37.2.381

[19] Steinert S, White DM, Zou Y, Shay JW, Wright WE. Telomere biology and cellular aging in nonhuman primate cells. *Experimental cell research*. 2002;272(2):146–52. . 11777339 10.1006/excr.2001.5409

[20] McGill HC Jr, Carey KD, McMahan CA, Marinez YN, Cooper TE, Mott GE, et al Effects of two forms of hypertension on atherosclerosis in the hyperlipidemic baboon. *Arteriosclerosis*. 1985;5(5):481–93. . 3899070 10.1161/01.atv.5.5.481

[21] Bronikowski AM, Alberts SC, Altmann J, Packer C, Carey KD, Tatar M. The aging baboon: comparative demography in a non-human primate. *Proc Natl Acad Sci U S A*. 2002;99(14):9591–5. . 12082185 10.1073/pnas.142675599 PMC123185

[22] Yeung KR, Lind JM, Heffernan SJ, Sunderland N, Hennessy A, Makris A. Comparison of indirect and direct blood pressure measurements in baboons during ketamine anaesthesia. *Journal of Medical Primatology*. 2014;43(4):217–24. 10.1111/jmp.12113 24628125

[23] Kurtz TW, Griffin KA, Bidani AK, Davisson RL, Hall JE. Recommendations for blood pressure measurement in humans and experimental animals: part 2: blood pressure measurement in experimental animals: a statement for professionals from the Subcommittee of Professional and Public Education of the American Heart Association Council on High Blood Pressure Research. *Arteriosclerosis*, *Thrombosis*, *and Vascular Biology*. 2005;25(3):e22–33. . 15731483 10.1161/01.ATV.0000158419.98675.d7

[24] Cawthon RM. Telomere measurement by quantitative PCR. *Nucleic acids research*. 2002;30(10):e47 . 12000852 10.1093/nar/30.10.e47 PMC115301

[25] Livak KJ, Schmittgen TD. Analysis of relative gene expression data using real-time quantitative PCR and the 2(-Delta Delta C(T)) Method. *Methods*. 2001;25(4):402–8. . 11846609 10.1006/meth.2001.1262

[26] Cefalu WT, Wagner JD. Aging and atherosclerosis in human and nonhuman primates. *Age*. 1997;20(1):15–28. 10.1007/s11357-997-0002-4 23604288 PMC3456081

[27] Hansen BC, Bodkin NL. Primary prevention of diabetes mellitus by prevention of obesity in monkeys. *Diabetes*. 1993;42(12):1809–14. . 8243827 10.2337/diab.42.12.1809

[28] Bailey JF, Fields AJ, Liebenberg E, Mattison JA, Lotz JC, Kramer PA. Comparison of vertebral and intervertebral disc lesions in aging humans and rhesus monkeys. *Osteoarthritis and cartilage / OARS*, *Osteoarthritis Research Society*. 2014;22(7):980–5. 10.1016/j.joca.2014.04.027 24821664 PMC4105267

[29] Beltran WA, Vanore M, Ollivet F, Nemoz-Bertholet F, Aujard F, Clerc B, et al Ocular findings in two colonies of gray mouse lemurs (Microcebus murinus). *Veterinary ophthalmology*. 2007;10(1):43–9. . 17204127 10.1111/j.1463-5224.2007.00491.x

[30] Darusman HS, Gjedde A, Sajuthi D, Schapiro SJ, Kalliokoski O, Kristianingrum YP, et al Amyloid Beta1-42 and the Phoshorylated Tau Threonine 231 in Brains of Aged Cynomolgus Monkeys (Macaca fascicularis). *Frontiers in aging neuroscience*. 2014;6:313 10.3389/fnagi.2014.00313 25426069 PMC4225838

[31] Zheng HY, Zhang MX, Pang W, Zheng YT. Aged Chinese rhesus macaques suffer severe phenotypic T- and B-cell aging accompanied with sex differences. *Experimental gerontology*. 2014;55:113–9. 10.1016/j.exger.2014.04.004 24746513

[32] Burt VL, Whelton P, Roccella EJ, Brown C, Cutler JA, Higgins M, et al Prevalence of hypertension in the US adult population. Results from the Third National Health and Nutrition Examination Survey, 1988–1991. *Hypertension*. 1995;25(3):305–13. . 7875754 10.1161/01.hyp.25.3.305

[33] Rodriguez BL, Labarthe DR, Huang B, Lopez-Gomez J. Rise of blood pressure with age. New evidence of population differences. *Hypertension*. 1994;24(6):779–85. . 7995637 10.1161/01.hyp.24.6.779

[34] Stoner L, Young JM, Fryer S. Assessments of arterial stiffness and endothelial function using pulse wave analysis. *International journal of vascular medicine*. 2012;2012:903107 10.1155/2012/903107 22666595 PMC3361177

[35] Vlachopoulos C, O'Rourke M. Genesis of the normal and abnormal arterial pulse. *Current problems in cardiology*. 2000;25(5):303–67. . 10822214 10.1067/mcd.2000.104057

[36] O'Rourke MF, Pauca A, Jiang XJ. Pulse wave analysis. *British journal of clinical pharmacology*. 2001;51(6):507–22. . 11422010 10.1046/j.0306-5251.2001.01400.x PMC2014492

[37] Oliver JJ, Webb DJ. Noninvasive assessment of arterial stiffness and risk of atherosclerotic events. *Arteriosclerosis*, *Thrombosis*, *and Vascular Biology*. 2003;23(4):554–66. . 12615661 10.1161/01.ATV.0000060460.52916.D6

[38] Smucny DA, Allison DB, Ingram DK, Roth GS, Kemnitz JW, Kohama SG, et al Changes in blood chemistry and hematology variables during aging in captive rhesus macaques (Macaca mulatta). *Journal of Medical Primatology*. 2001;30(3):161–73. . 11515672 10.1111/j.1600-0684.2001.tb00005.x

[39] Smucny DA, Allison DB, Ingram DK, Roth GS, Kemnitz JW, Kohama SG, et al Changes in blood chemistry and hematology variables during aging in captive rhesus macaques (Macaca mulatta). J Med Primatol 30:161–173, 2001. *Journal of Medical Primatology*. 2004;33(1):48–54. . 15061733 10.1111/j.1600-0684.2003.00052.x

[40] Visser M, Kritchevsky SB, Newman AB, Goodpaster BH, Tylavsky FA, Nevitt MC, et al Lower serum albumin concentration and change in muscle mass: the Health, Aging and Body Composition Study. *Am J Clin Nutr*. 2005;82(3):531–7. . 16155264 10.1093/ajcn.82.3.531

[41] Bourdel-Marchasson I, Laksir H, Puget E. Interpreting routine biochemistry in those aged over 65 years: a time for change. *Maturitas*. 2010;66(1):39–45. 10.1016/j.maturitas.2010.02.004 20197224

[42] Bohnen N, Degenaar CP, Jolles J. Influence of age and sex on 19 blood variables in healthy subjects. *Zeitschrift fur Gerontologie*. 1992;25(5):339–45. . 1441715

[43] Chapman KM, Ham JO, Pearlman RA. Longitudinal assessment of the nutritional status of elderly veterans. *The journals of gerontology Series A*, *Biological sciences and medical sciences*. 1996;51(4):B261–9. . 8680990 10.1093/gerona/51a.4.b261

[44] Fraser CG. Age-related changes in laboratory test results. Clinical implications. *Drugs & aging*. 1993;3(3):246–57. . 8324300 10.2165/00002512-199303030-00006

[45] Tietz NW, Shuey DF, Wekstein DR. Laboratory values in fit aging individuals—sexagenarians through centenarians. *Clinical Chemistry*. 1992;38(6):1167–85. . 1596990

[46] Murray CE, Warnes DM, Ballard FJ, Tomas FM. Creatinine excretion as an index of myofibrillar protein mass in dystrophic mice. *Clinical science*. 1981;61(6):737–41. . 7297035 10.1042/cs0610737

[47] Grundy SM, Bilheimer D, Chait A, Clark LT, Denke M, Havel RJ, et al Summary of the second report of the National Cholesterol Education Program (NCEP) Expert Panel on Detection, Evaluation, and Treatment of High Blood Cholesterol in Adults (Adult Treatment Panel II). *JAMA*: *the journal of the American Medical Association*. 1993;269(23):3015–23. . 8501844

[48] Schaefer EJ, Lichtenstein AH, Lamon-Fava S, McNamara JR, Ordovas JM. Lipoproteins, nutrition, aging, and atherosclerosis. *The American journal of clinical nutrition*. 1995;61(3 Suppl):726S–40S. . 7879744 10.1093/ajcn/61.3.726S

[49] Baerlocher GM, Mak J, Roth A, Rice KS, Lansdorp PM. Telomere shortening in leukocyte subpopulations from baboons. *Journal of leukocyte biology*. 2003;73(2):289–96. . 12554806 10.1189/jlb.0702361

[50] Rufer N, Brummendorf TH, Kolvraa S, Bischoff C, Christensen K, Wadsworth L, et al Telomere fluorescence measurements in granulocytes and T lymphocyte subsets point to a high turnover of hematopoietic stem cells and memory T cells in early childhood. *The Journal of experimental medicine*. 1999;190(2):157–67. . 10432279 10.1084/jem.190.2.157 PMC2195579

[51] Bansal N, Whooley MA, Regan M, McCulloch CE, Ix JH, Epel E, et al Association between kidney function and telomere length: the heart and soul study. *American journal of nephrology*. 2012;36(5):405–11. 10.1159/000343495 23108000 PMC3552638

[52] Blackburn EH. Structure and function of telomeres. *Nature*. 1991;350(6319):569–73. . 1708110 10.1038/350569a0

[53] Blackburn EH. Telomere states and cell fates. *Nature*. 2000;408(6808):53–6. . 11081503 10.1038/35040500

[54] Fyhrquist F, Saijonmaa O, Strandberg T. The roles of senescence and telomere shortening in cardiovascular disease. *Nature reviews Cardiology*. 2013;10(5):274–83. 10.1038/nrcardio.2013.30 23478256

