# Supplementary material for: A Cross-Sectional Study of Ageing and Cardiovascular Function over the Baboon Lifespan
Source: PLoS One. 2016 Jul 18;11(7):e0159576. doi: 10.1371/journal.pone.0159576 (PMC4948874; doi:10.1371/journal.pone.0159576)
Supplement: S1 Table — (DOCX) [file pone.0159576.s001.docx]

**S1 Table.** Correlation between cardiovascular and biochemical parameters with age, stratified by sex

| **Variable** |  | **Pearson correlation coefficient** | **Adj. R^2^** | **P value** |
| --- | --- | --- | --- | --- |
| **Weight** | Male | 0.64 | 0.10 | <0.001 |
|  | Female | 0.57 | 0.31 | <0.001 |
| **Systolic blood pressure** | Male | 0.35 | 0.10 | 0.008 |
|  | Female | 0.64 | 0.40 | <0.001 |
| **Diastolic blood pressure** | Male | 0.65 | 0.40 | <0.001 |
|  | Female | 0.69 | 0.46 | <0.001 |
| **Heart rate** | Male | -0.26 | 0.05 | 0.04 |
|  | Female | -0.02 | -0.02 | 0.42 |
| **Core augmented index** | Male | 0.43 | 0.17 | 0.001 |
|  | Female | 0.40 | 0.15 | 0.001 |
| **Av. core pulse pressure** | Male | 0.47 | 0.20 | <0.001 |
|  | Female | 0.62 | 0.37 | <0.001 |
| **Cholesterol** | Male | -0.40 | 0.14 | 0.003 |
|  | Female | -0.18 | 0.01 | 0.10 |
| **Creatinine** | Male | 0.71 | 0.49 | <0.001 |
|  | Female | 0.58 | 0.33 | <0.001 |
| **Triglycerides** | Male | 0.15 | 0.001 | 0.15 |
|  | Female | 0.14 | 0.001 | 0.16 |
| **Urea** | Male | 0.21 | 0.02 | 0.08 |
|  | Female | 0.45 | 0.19 | <0.001 |
| **Serum sodium** | Male | -0.43 | 0.17 | 0.001 |
|  | Female | -0.12 | -0.003 | 0.19 |
| **Serum Albumin** | Male | -0.35 | 0.10 | 0.01 |
|  | Female | 0.09 | -0.01 | 0.26 |
| **Urinary protein** | Male | -0.21 | 0.02 | 0.08 |
|  | Female | 0.36 | 0.11 | 0.002 |
| **Urinary micro-albumin** | Male | -0.09 | -0.01 | 0.26 |
|  | Female | 0.29 | 0.07 | 0.01 |
